# Lymphocytic Pleural Effusion and an Enzyme Involved in Purine Metabolism: A Tertiary Care Experience in Karachi, Pakistan

**DOI:** 10.7759/cureus.4069

**Published:** 2019-02-13

**Authors:** Rashid Naseem Khan, Syed Ijlal Ahmed, Syedhh Fatima Kausar, Farhana Saba, Sadia Din, Zia Ud Deen, Ali Shah

**Affiliations:** 1 Internal Medicine, Darul Sehat Hospital, Karachi, PAK; 2 Neurology, Liaquat National Hospital and Medical College, Karachi, PAK; 3 Internal Medicine, Darul Sehat Hospital, Karachi , PAK; 4 Internal Medicine, Dow University of Health Sciences, Karachi, USA; 5 Surgery, Dow University of Health Sciences, Karachi, PAK

**Keywords:** tuberculous, purine metabolism, malignancy

## Abstract

Background: The levels of adenosine deaminase (ADA) are increased in tubercular pleural effusion and its determination has acquired popularity as a diagnostic test which is inexpensive and is readily accessible. Pleural fluid ADA showed sensitivity (86.36%), specificity (61.54%), diagnostic accuracy (80.70%), positive predictive value (88.37%), and negative predictive value (82.42%) confirmed by pleural biopsy as a gold standard.

Methodology: Our study was a prospective cross-sectional study which was conducted for three years at a tertiary care center in Karachi, Pakistan. The data were collected and analyzed using IBM statistics SPSS vs21.

Results: There were 52 patients included in our study. Twenty one were males and thirty one were females. Most patients presented with shortness of breath. There was a significant association found between raised ADA levels and pulmonary tuberculosis (p < 0.05). The ADA levels are 12 times more likely to be raised in tubercular pleural effusion.

Conclusion: The ADA level is an important marker for diagnosis of pulmonary tuberculosis in lymphocytic pleural effusion. It is a convenient and an inexpensive method. The ADA levels assessment is economical when compared to other diagnostic methods.

## Introduction

Pleural effusion is a common medical condition with many possible underlying etiologies. Neutrophilic predominant exudative effusions are due to acute processes, e.g., pneumonia or acute pulmonary embolism [1], whereas, lymphocytic effusions have a much longer list of differential diagnoses [2]. However, in areas with high incidence of tuberculosis (TB), pleural TB and malignancy are the most likely causes of a lymphocytic pleural effusion [3-4].

Adenosine deaminase (ADA) is an enzyme involved in purine metabolism which catalyzes the conversion of adenosine to inosine and plays an important role in the differentiation of lymphoid cells. Its activity is high in diseases in which cellular immunity is stimulated.

Conventionally, the diagnosis of tubercular pleural effusion is made based on the clinical data, biochemical, and microscopic examination of pleural fluid as exudates and contains high count of lymphocytes which is nonspecific. Other methods are Zeil Nelson staining and culture of pleural fluid which lack sensitivity, i.e., 10%-40% and 8%-49%, respectively. At the same time culture identification takes a long time, i.e., four to six weeks. Pleural biopsy is an invasive procedure with a sensitivity of 50%-80% and not a routine test [5].

The levels of ADA are increased in tubercular pleural effusion and its determination has acquired popularity as a diagnostic test which is noninvasive, not expensive, and is readily accessible.

Pleural fluid ADA showed sensitivity (86.36%), specificity (61.54%), diagnostic accuracy (80.70%), positive predictive value (88.37%), and negative predictive value (82.42%) confirmed by pleural biopsy as a gold standard [6].

Our study is aimed to assess the diagnostic importance of ADA in tubercular pleural effusion from Pakistan. According to the best of our knowledge much less data are available in medical literature from developing world regarding ADA levels in pulmonary TB.

## Materials and methods

Our study was a prospective comparative cross-sectional observational study conducted at a tertiary care center in Karachi, Pakistan. The duration of our study was three years. The sample size was calculated using the World Health Organization (WHO) sample size calculator. The sampling technique of our study was nonprobability. Informed consent for participation in the study was taken from the patients. The data were collected by post graduate trainees of the Department of Medicine. The variables of our study include the demographics of patients (age, gender, medical record number, and duration of stay in hospital), clinical presentation of patient, co-morbid of the patients, final diagnosis, cytology results of pleural fluid, and ADA levels in pleural fluid of patients.

The inclusion criterion of our study was, all the patients visiting the clinics of the Department of Medicine in Darul Sehat Hospital with suspicion of pulmonary TB or malignancy on the basis of history and examination findings.

The exclusion criterion of our study was the exclusion of patients who presented without suspicion of TB or malignancy and patients requiring surgical intervention.

The data were recorded by post graduate trainees of Medicine. The data were analyzed for any descriptive statistics. Data were also analyzed for any association or correlation among variables using chi-square test, independent t-test, and correlation testing.

All ethical considerations were taken during the study.

## Results

**Figure 1:**
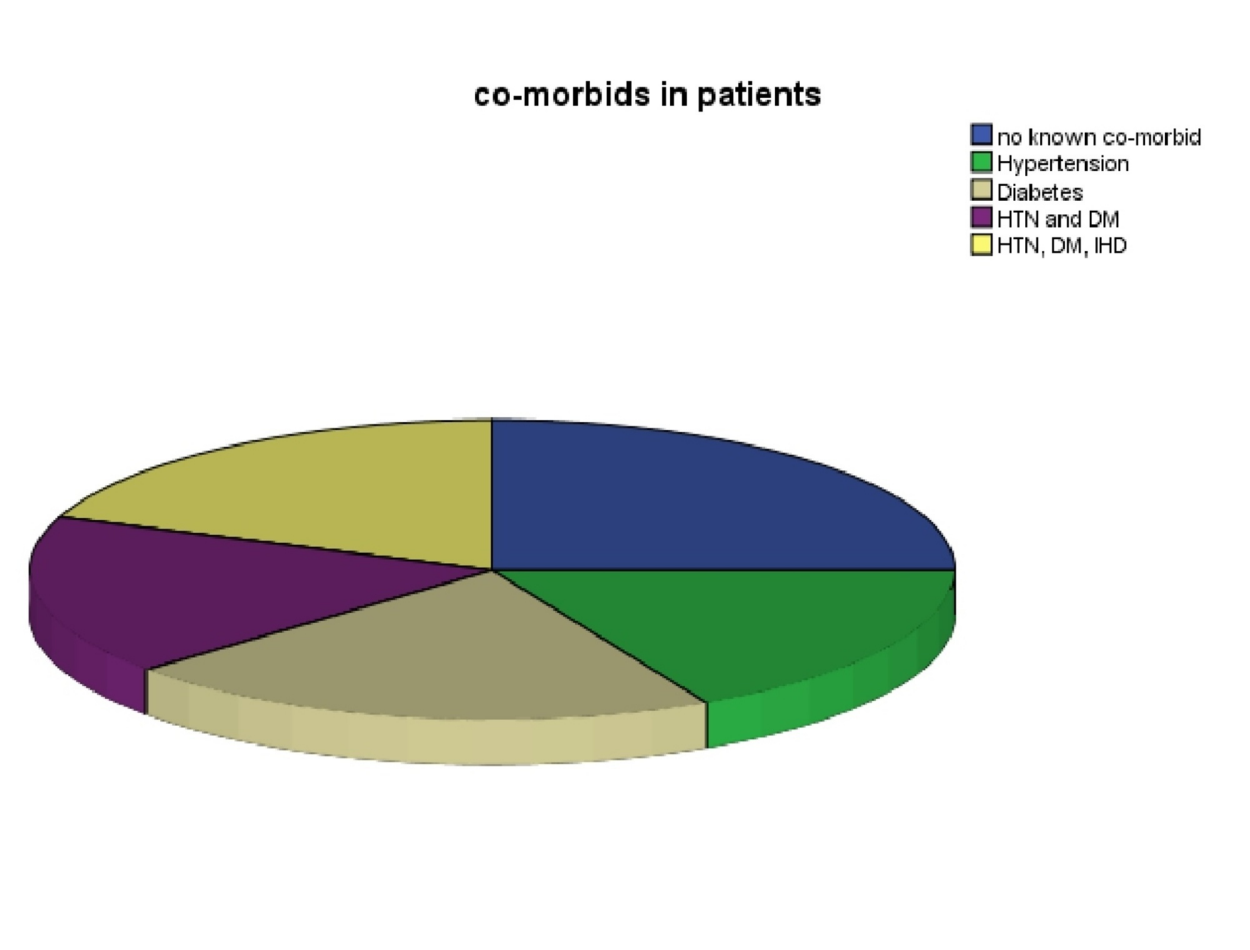
Co-morbids of patients. The figure shows the co-morbids of study population, e.g., hypertension (HTN), diabetes mellitus (DM), and ischemic heart disease (IHD).

Fifty two patients were included in our study—twenty one were males and thirty one were females. The mean age of patients was 54 ± 16 years. The mean duration of patients stay in hospital was 8.8 ± 6.1 days. The most common presentation of patients was shortness of breath (59.1%) and the most common examination finding was bilateral crepitations in the chest (38.5%). The co-morbids of patients are shown in Figure 1.

The final diagnoses in patients with lymphocytic pleural effusion patients were TB (88.5%) and pulmonary malignancy (11.5%).

In 88.5% patients we found lymphocytosis without malignant cells. The mean ADA levels in patients were 34.5 ± 15.3 IU/L. Among 46 patients with tuberculous effusion, 33 patients had raised ADA levels (>33 IU/L) while 13 had normal ADA levels (Table 1).

**Table 1:** Relationship of adenosine deaminase (ADA) levels with pulmonary tuberculosis (TB) and malignancy.

Adenosine deaminase (ADA) levels in lymphocytic pleural effusion				
		Raised	Normal	Total
Final diagnosis in patients	Pulmonary tuberculosis (TB)	33	13	46
	Pulmonary malignancy	1	5	6
Total		34	18	52

The variables were also observed for any significant association using chi-square test. There was a significant association found between raised ADA levels and pulmonary TB (p = 0.015).

The binary logistic model was also performed to ascertain the effects of pulmonary TB on the likelihood of raised ADA levels in patients with lymphocytic pleural effusions. The model was statistically significant (p < 0.05). The model explained 17.1% variance and correctly classified 73.1% of the cases. The ADA levels are 12 times likely to be raised in pulmonary TB.

## Discussion

When a patient presents with new pleural effusion, the diagnosis of TB pleuritis should be considered [7]. The patient is at risk for developing pulmonary or extrapulmonary TB if the diagnosis is not made. Between 3% and 25% of patients with TB will have TB pleuritis [7]. The incidence of TB pleuritis is higher in patients who are human immunodeficiency virus (HIV)-positive. Pleural fluid is an exudate that usually has a predominance of lymphocytes. The easiest way to diagnose TB pleuritis in a patient with lymphocytic pleural effusion is to demonstrate a pleural fluid ADA level above normal level [7].

The results of our study demonstrate that it is possible to establish the diagnosis of TB pleural effusion from clinical data and pleural fluid analysis in suspected cases in regions with high prevalence of TB using ADA levels.

Pakistan accounts for 61% of the TB burden in the WHO Eastern Mediterranean Region. In our setting, TB is the most common cause of pleural effusions (88.5%) among the patients admitted. Most of the patients presented with shortness of breath (51.9%), fever and cough (26.9%), and the most common examination finding was bilateral crackles (38.5%). These symptoms were similar to presentations and examination findings in other studies [8-9].

One international study from India [10] discussed that the most common cause of pleural effusion was malignancy which is in contrast to our study where the most common cause was TB. In their study the mean ADA level was 28 which is lower than the mean of our study which is 35. The TB pleural effusion was predominant in men while in our setting it was found more predominant in women. Pleural effusion fluid analysis in our study showed lymphocytosis without malignant cells (88.5%) in patients with TB which is higher than an international study [11] which was 85%. Most of the TB pleural effusion patients had raised ADA levels, making it a better diagnostic tool in suspected cases of pleural TB.

A study from India suggested pleural fluid ADA levels are highly sensitive for TB pleural effusions. ADA is diagnostic even in HIV positive patients with TB pleural effusion. The ADA levels easily differentiate TB pleural effusion from parapneumonic, malignant, pancreatic, and amoebic pleural effusions [12].

Another study from India reported that all patients with TB pleural effusion had elevated ADA levels and there was a statistical significant association (p < 0.05) of ADA levels in differentiating TB pleural effusion from non-TB pleural effusion [13].

A study from Georgia concluded that pleural fluid ADA remains useful in diagnosing TB pleural effusion. The analysis of ADA levels can be done simply, quickly, and cheaply. In their study the ADA levels were significantly higher in TB pleural effusion patients [14].

In the most recent meta-analysis of studies investigating the use of pleural fluid ADA for diagnostic evaluation in pleural TB, 63 studies have been analyzed and showed high sensitivity and specificity [15]. Several reports have suggested that raised pleural fluid ADA level predicts TB pleuritis with a sensitivity of 90%-100% and a specificity of 89%-100% [16]. The positive predictive value of a test is higher in regions with higher prevalence of a particular disease [17]. Pleural fluid ADA in countries with higher prevalence of TB makes it economically a better option.

Even though the gold standard for the diagnosis of TB pleuritis remains the detection of mycobacterium TB in pleural fluid, or pleural biopsy specimens, either by microscopy and/or culture, or the histological demonstration of caseating granulomas in the pleura along with acid fast bacilli [18], pleural ADA testing being noninvasive and inexpensive is an invaluable tool for rapid and accurate diagnosis in developing countries like Pakistan.

The limitation of our study includes the relatively smaller sample size. In future similar studies with larger sample size should be done from Pakistan because Pakistan is an endemic country for TB.

## Conclusions

Adenosine deaminase level is an important marker for diagnosis of pulmonary TB in lymphocytic pleural effusion. It is a convenient and an inexpensive method. The ADA levels assessment is economical when compared to other diagnostic methods.
